# Postponing evolution: why are we choosing to ignore the need for a digital transformation in pathology?

**DOI:** 10.1007/s00428-023-03714-3

**Published:** 2023-12-04

**Authors:** Catarina Eloy

**Affiliations:** 1 Pathology Laboratory, Institute of Molecular Pathology and Immunology of University of Porto (IPATIMUP), Rua Júlio Amaral de Carvalho 45, 4200-135 Porto, Portugal; 2 Pathology Department, Medical Faculty of University of Porto, Porto, Portugal

**Keywords:** Digital pathology, Digital transformation, Evolution, Computational pathology, Pathology laboratory

For some years now, practical evidence on the benefits of using whole slide images (WSIs) for primary diagnosis in pathology has been consistently reported [1–3]. It is also clear that, besides the announced benefits, WSI usage is far from being ubiquitous, representing the exception, rather than the rule [4].

During the early days, the possibility of generating a file from a glass slide using a rudimentary scanner gathered the enthusiasm of a restricted group of pathologists specifically cultivated in informatic sciences. The plasticity of WSIs, namely for distant sharing (telepathology), was admirable [5]. Then, the technology progressed and a new generation of scanners, robust enough to be used in the clinical setting for primary diagnosis, appeared in the market, democratizing high-quality and fast scanning. Digital pathology started to be heard as a common idea. The meaning of the, so called, digital pathology (an unfortunate designation since there are no “digital” diseases) has evolved from a scanner-centric concept to a holistic process that challenges the management of the laboratory. Apparently, in the process of transforming a glass slide into a WSI, more is needed than just a scanner. Lessons learned from successful and sustainable early adopters of the digital workflow [1–3] highlight the need for a broad intervention in the laboratory functioning. This intervention, not restricted at all to scanning, includes all laboratory workstations as well as the strict control of pre- and postanalytic variables, to achieve the best quality glass slide to be scanned [6]. This holistic concept of digital pathology pushes pathology into a transversal movement, crossing all sorts of professions inside and outside the health care environment, which is the digital transformation.

Why is this scanner-centric concept of digital pathology not enough and why do we need a digital transformation instead? There are well-known fragilities in the tissue processing methodology used every day in pathology laboratories. Tissue processing encompasses activities that are predominantly non-standardized, manual, and slow, prone to all sorts of errors [7]. There are many examples in our daily practice of frequent situations where the laboratory product (the glass slide with a tissue preparation) is not satisfactory for an observation with precision diagnosis purposes. Mislabelling, tissue floaters, thick/uneven cut thickness, unstable staining, and poor coverslipping are some of the causes of inadequate laboratory product that may variably occur in each institution. Since scanning suboptimal glass slides generates poor quality WSIs, it is probably not appropriate to start scanning before optimizing the quality of the laboratory product. Nearly 45% of pathology laboratories with scanners in Europe and Asia claim that one of the major obstacles to the digital primary diagnosis is still the quality of the WSIs [8].

The digital transformation of the pathology laboratory is, in fact, a progressive evolution towards automation and standardization that should culminate with the introduction of scanning and, afterwards, the implementation of computational pathology. It includes the need of a reliable laboratory information system that tracks the samples preventing losses and mislabelling, connects instruments contributing to real-time integrated data, allows electronic and precise records, helps organizing human resources according to the workload, supports the capture of digital images, and more, according to the specific needs of every institution. It is easy to understand that, even not generating an immediate financial return on the investment, the digital transformation results in major gains on quality and efficiency of the workflow. On the other hand, only a strong investment in quality control measures allows the production of WSIs adequate for precision diagnosis. These two effects, connecting reciprocally the digital transformation and quality, can be understood as two sides of the same coin, increasing (or decreasing) in parallel. It is never enough to emphasize that both quality and efficiency, with full respect by turnaround time, are the main contributors to the reputation of the pathologist.

Difficulties related to the unavailability of disruptive laboratory instruments or laboratory information systems dedicated to the modern tissue processing represent one of the major obstacles to the digital transformation. Industry leaders in pathology instruments and software are key players in the success of the global digital transformation. The frequent stagnation in the investment on updated instruments that occur in some institutions and the consistent low value attributed to pathology departments do not wield a stimulation effect in industry regarding breakthroughs. It is easy to understand that this circle of low investment tends to deaccelerate evolution in pathology, a tendency that may be disrupted by each institution supported by adequate reimbursement policies.

Other factors that may contribute to the failure of digital transformation are easy to anticipate. The digital transformation depends on financial resources, requires informatic expertise to its implementation, and, importantly, requires persistent action, from both pathologists and laboratory technicians. Making the beginning of the digital transformation to depend only on the scanner acquisition and/or informatic support is to postpone the adoption. The digital transformation is a laborious and time-consuming process that requires pathologists to adjust together with laboratory technicians, the most efficient and automatized design of each workstation (accessioning, macroscopy station, embedding, cutting, staining, coverslipping, scanning, reporting, complementary techniques, and others) as well as establishing the digital storage design and policy according to the local regulations. Working together with laboratory technicians at the bench has become a strange task to many pathologists. Pathology has evolved based on the growing complexity of the diagnostic process that dominates the scene, asphyxiating the interest on the immutable technical environment. An active approximation of the pathologist to the laboratory life is not easily substituted by the intervention of other groups that will contribute to other steps of the global process of transformation (informatic experts, molecular biologists, managers, others). So, admitting the possibility of available funds for the digital transformation, the solution is still not at hand for all institutions if we keep in mind that world-wide pathology is suffering from workforce shortage.

The optimism associated with the usage of computational pathology tools, namely artificial intelligence (AI), is growing among pathologists [9]. Generated in-house or commercially available, computational pathology tools have the promise to decrease the pathologist’s workload, to facilitate the execution of repetitive tasks such as identification and quantification, and to predict outcomes, among other announced benefits. These seductive AI tools may be used in WSI files and have been adopted by some pathology laboratories prior to the digital transformation. It is not unusual to encounter a scenario where only the glass slides to be evaluated by AI are scanned. These selected glass slides are often prepared as the remaining ones in the daily routine, without any special quality stress. Nevertheless, the performance of AI tools is heavily dependent on the quality of the WSI, and the quality of the respective glass slides as well. Poor quality WSIs may introduce serious bias into the output of the AI tools [10]. Other practical factors related with AI tools usage such as difficulties in integration with the laboratory information system, sustained validation procedures, decisions on the reporting of conflicting data, and long-term preservation of records may intensify the improper usage of this technology. Ultimately, the adoption of computational pathology tools without a previous solid digital transformation is an unacceptable short cut that denies the possibility to elevate pathology into another stage and to definitely postpone evolution of pathology.

Here is presented the ultimate challenge to pathology evolution that needs to be embraced by all of us, as part of a cohesive and responsible community, that purges for the best patient care.

## Data Availability

No data was generated.

## References

[1] Eloy C, Vale J, Curado M, Polonia A, Campelos S, Caramelo A, Sousa R, Sobrinho-Simoes M (2021) Digital pathology workflow implementation at IPATIMUP. Diagnostics (Basel) 11. 10.3390/diagnostics11112111 10.3390/diagnostics11112111 PMC8620597 34829458

[2] Fraggetta F, Caputo A, Guglielmino R, Pellegrino MG, Runza G, L'Imperio V (2021) A survival guide for the rapid transition to a fully digital workflow: the “caltagirone example”. Diagnostics (Basel) 11. 10.3390/diagnostics11101916 10.3390/diagnostics11101916 PMC8534326 34679614

[3] Retamero JA, Aneiros-Fernandez J, Del Moral RG (2020) Complete digital pathology for routine histopathology diagnosis in a multicenter hospital network. Arch Pathol Lab Med 144:221–228. 10.5858/arpa.2018-0541-OA 31295015 10.5858/arpa.2018-0541-OA

[4] Griffin J, Treanor D (2017) Digital pathology in clinical use: where are we now and what is holding us back? Histopathology 70:134–145. 10.1111/his.12993 27960232 10.1111/his.12993

[5] Farahani N, Pantanowitz L (2015) Overview of telepathology. Surg Pathol Clin 8:223–231. 10.1016/j.path.2015.02.018 26065796 10.1016/j.path.2015.02.018

[6] Fraggetta F, L'Imperio V, Ameisen D, Carvalho R, Leh S, Kiehl TR, Serbanescu M, Racoceanu D, Della Mea V, Polonia A, Zerbe N, Eloy C (2021) Best practice recommendations for the implementation of a digital pathology workflow in the anatomic pathology laboratory by the European Society of Digital and Integrative Pathology (ESDIP). Diagnostics (Basel) 11. 10.3390/diagnostics11112167 10.3390/diagnostics11112167 PMC8623219 34829514

[7] Zarbo RJ (2022) The unsafe archaic processes of tissue pathology. Am J Clin Pathol 158:4–7. 10.1093/ajcp/aqac018 35229867 10.1093/ajcp/aqac018

[8] Pinto DG, Bychkov A, Tsuyama N, Fukuoka J, Eloy C (2023) Exploring the adoption of digital pathology in clinical settings - Insights from a cross-continent study medRxiv:2023.2004.2003.23288066 10.1101/2023.04.03.23288066

[9] Berbis MA, McClintock DS, Bychkov A, Van der Laak J, Pantanowitz L, Lennerz JK, Cheng JY, Delahunt B, Egevad L, Eloy C, Farris AB 3rd, Fraggetta F, Garcia Del Moral R, Hartman DJ, Herrmann MD, Hollemans E, Iczkowski KA, Karsan A, Kriegsmann M, Salama ME, Sinard JH, Tuthill JM, Williams B, Casado-Sanchez C, Sanchez-Turrion V, Luna A, Aneiros-Fernandez J, Shen J (2023) Computational pathology in 2030: a Delphi study forecasting the role of AI in pathology within the next decade. EBioMedicine 88:104427. 10.1016/j.ebiom.2022.104427 36603288 10.1016/j.ebiom.2022.104427 PMC9823157

[10] Schömig-Markiefka B, Pryalukhin A, Hulla W, Bychkov A, Fukuoka J, Madabhushi A, Achter V, Nieroda L, Büttner R, Quaas A, Tolkach Y (2021) Quality control stress test for deep learning-based diagnostic model in digital pathology. Mod Pathol. 10.1038/s41379-021-00859-x 34168282 10.1038/s41379-021-00859-x PMC8592835

